# Getting acquainted: First steps for child-robot relationship formation

**DOI:** 10.3389/frobt.2022.853665

**Published:** 2022-09-15

**Authors:** Mike E. U. Ligthart, Mark A. Neerincx, Koen V. Hindriks

**Affiliations:** ^1^ Social AI, Vrije Universiteit Amsterdam, Amsterdam, Netherlands; ^2^ Interactive Intelligence, Delft University of Technology, Delft, Netherlands; ^3^ Perceptual & Cognitive Systems, TNO, Soesterberg, Netherlands

**Keywords:** child-robot interaction, human-robot interaction, social robots, getting acquainted, self-disclosure, relationship formation, user study

## Abstract

In this article we discuss two studies of children getting acquainted with an autonomous socially assistive robot. The success of the first encounter is key for a sustainable long-term supportive relationship. We provide four validated behavior design elements that enable the robot to robustly get acquainted with the child. The first are five conversational patterns that allow children to comfortably self-disclose to the robot. The second is a reciprocation strategy that enables the robot to adequately respond to the children’s self-disclosures. The third is a ‘how to talk to me’ tutorial. The fourth is a personality profile for the robot that creates more rapport and comfort between the child and the robot. The designs were validated with two user studies (*N*_1_ = 30, *N*_2_ = 75, 8–11 years. o. children). The results furthermore showed similarities between how children form relationships with people and how children form relationships with robots. Most importantly, self-disclosure, and specifically how intimate the self-disclosures are, is an important predictor for the success of child-robot relationship formation. Speech recognition errors reduces the intimacy and feeling similar to the robot increases the intimacy of self-disclosures.

## 1 Introduction

More and more socially assistive robot (SAR) applications are being developed to serve as (mental) health interventions for children (Kabacińska et al., 2021). It is the social interaction that these SARs aim to offer that stands at the core of the intervention (Feil-Seifer and Mataric, 2005). We are, for example, developing a social robot companion (a Nao robot we call Hero) for pediatric oncology patients (Ligthart et al., 2018b). Our main objective is to contribute to stress reduction. For that purpose we developed a narrative (i.e., story-rich) conversation with Hero that offers children an engaging distraction from stressful situations during the treatment (e.g., placement of an IV catheter) Ligthart et al. (2020), Ligthart et al. (2021).

To move beyond a single session intervention that relies primarily on the novelty effect (Kabacińska et al., 2021), we need to design a grounded and satisfying social interaction that kick-starts the supportive relationship we are looking for. A lot of research is done exploring different types of interventions with various robot platforms (e.g., see the scoping review of Kabacińska et al. (2021)), however not much research exists that studies how to robustly facilitate a grounded and satisfying social interaction between children and robots.

With this article we intent to contribute to filling that gap in knowledge. Firstly, we increase our understanding of how to ground a social interaction between children and robots and how to foster a supportive relationship. Secondly, we provide reusable and concrete behavior designs for an autonomously operating robot. Thirdly, we validate the theoretical operationalizations and designs we put forward in this article with two user studies.

Socially assistive robots need to operate in a long-term context with multiple sessions. The first encounter and the resulting first impressions have an important impact on the long-term success of the interaction (Berscheid and Regan, 2005; Paetzel et al., 2020) and relationship formation (Bergmann et al., 2012; Sprecher et al., 2015). The more accurate the first impressions are, the more positive the impact (Human et al., 2013). Enabling the robot and the child to properly get acquainted is critical for facilitating a grounded interaction (Ligthart et al., 2018a; Ligthart et al., 2019a) that is sustainable long-term (Ligthart et al., 2019b).

In this article we focus on the getting acquainted interaction and in particular on the role of reciprocal self-disclosure. We specified four different behavioral design elements. The first is a set of five conversational patterns that elicit children to self-disclose. The second is a tutorial that teaches children how to talk to the robot. The third are two different reciprocation strategies for the robot. The fourth are two distinct behavioral profiles for the robot that differ in their arousal level. The high arousal profile was meant to match with extraverted children and the low arousal profile with introverted children. All four design elements aim to contribute to the robot and the child getting acquainted autonomously and to foster the child-robot relationship.

To evaluate these aspects we ran two user studies. In the first user study (*N* = 30 8–10 years. o children) we compared the two reciprocation strategies. In the second user study (*N* = 75 8–11 years. o children) we validated the conversational patterns, the tutorial, and the arousal behavior profiles. To not create an additional burden or source of stress for the children in the hospital we involved healthy school children in testing the prototypes. This is an approach that is not uncommon when developing social robots for children with a special needs (Neerincx et al., 2019). The clinical trials are currently underway and will be reported in future work. As a benefit, the designs and results are more generically applicable and can be applied to a wide variety of domains.

The article is structured as follows. First, we compile a design foundation from related work and identify important design requirements. Then we discuss the specifications and the design rational of the four robot behavior designs. Then the methods and results of the two user studies are discussed. To conclude, we reflect on the designs and share the theoretical and practical lessons we learned about facilitating a grounded and satisfying autonomous child-robot conversation.

## 2 Design foundation

In order to design appropriate robot behaviors for a getting acquainted interaction we need to inform our design decisions with how people, and specifically children, get acquainted. In this section we provide a design foundation of related work. We discuss the social processes and key factors of getting acquainted, self-disclosure, reciprocation, relationship formation, and how the robot might facilitate those processes. Note that we do not aim to mirror these processes with the robot. Instead we try to identify interactional needs the children might have for the robot. We see these needs as requirements. How the robot meets these requirements need not be the same as how people do it. Better is to utilize the robot’s own strengths.

### 2.1 The first encounter and getting acquainted

The most natural way of getting to know someone is by striking up a conversation and talking about various topics freely (Svennevig, 2000). Following the social penetration theory, people slowly get to know each others’ interests, preferences, and stances on certain topics of an increasingly deepening degree (Altman and Taylor, 1973). This type of conversation is called an unstructured dyadic interaction (Ickes, 1983). Getting acquainted is such a common occurrence between people that we do not realize how complex that interaction really is. It seems to be an open interaction, however implicit social norms and biases shape the relationship formation process (Svennevig, 2000). For example, the more similar people perceive themselves to be to a new acquaintance, the more likely it is they become friends (Selfhout et al., 2009).

Following the uncertainty reduction theory, an important step in the getting acquainted process is taking away uncertainty about who the other is (Berger and Calabrese, 1975; Berscheid and Regan, 2005). Exchanging personal information, or self-disclosure, is a key mechanism for getting acquainted (Altman and Taylor, 1973; Vittengl and Holt, 2000). By self-disclosing people can take away that uncertainty (Sprecher et al., 2015) and discover similarities (Selfhout et al., 2009). Hence, our focus on supporting self-disclosure in the designs.

Although our focus lies on the conversational aspects of the getting acquainted interaction, we would like to point out that there is more to a first encounter. Factors like appearance (Bergmann et al., 2012; Paetzel et al., 2020; Reeves et al., 2020), non-verbal behavior (Bergmann et al., 2012; Calvo-Barajas et al., 2020), proxemics (Petrak et al., 2019), robot performance (Paetzel et al., 2020) play a vital role in the first impression of robots. Differences in all these factors also affect the perception of, and ultimately the relationship with, robots over time (Bergmann et al., 2012; Paetzel et al., 2020). Finally, we would also like to point out that there are different ways a first impression of a robot can be made. For example, for home robots the unboxing experience can be seen as a moment where a first impression is made (Lee et al., 2022).

### 2.2 Reciprocal self-disclosure

The importance of self-disclosure in relationship formation is not only evident in human-human relationships (Cozby, 1973; Derlega et al., 2008), but also in the relationships between children and artificial agents (Moon, 2000; Kanda et al., 2007; Kruijff-Korbayová et al., 2015; Burger et al., 2017; Kory Westlund et al., 2018). More self-disclosure generally increases liking (Cozby, 1972; Collins and Miller, 1994), closeness (Aron et al., 1997), positive affect (Vittengl and Holt, 2000), and rapport (Collins and Miller, 1994). All important indicators of a successful relationship formation (Derlega et al., 2008; Straten et al., 2020; van Straten et al., 2020).

One strategy for eliciting self-disclosure is by directly asking someone to self-disclose (e.g. already, albeit reluctantly^1^, shown by Weizenbaum (1966)’s ELIZA). By self-disclosing you put yourself at risk and make yourself vulnerable. Therefore, it is important that the partner reciprocates (Ehrlich and Graeven, 1971) and balances that risk. Reciprocation is not only a social norm of balancing risk, but also important for creating opportunities for both parties to self-disclose. Listening to someone self-disclose in itself already is important for relationship formation (Sprecher et al., 2013). This means that the robot must be able to self-disclose as well (Moon, 2000; Burger et al., 2017). The more the child learns about the robot, the more they have the desire to connect with the robot (Sprecher et al., 2013). Important design questions are how do these robot disclosures need to look like and how should the robot respond to a child’s self-disclosure?

The social penetration theory predicts that the self-disclosures start as basic biographical information and preferences about topics like food, music, clothing, etc. and becomes more intimate over time (Altman and Taylor, 1973). In response to the child self-disclosing the robot should at least acknowledge a self-disclosure (Birnbaum et al., 2016) and preferably reciprocate by disclosing something equivalent (Ehrlich and Graeven, 1971) with a matching level of intimacy (Burger et al., 2017).

### 2.3 Similarity

Not all people get acquainted in exactly the same way. A well studied concept in psychology is the similarity-attraction hypothesis. Research shows that people form relationships more easily if they have similar values (Burleson et al., 1992), attitudes (Byrne, 1961), personalities (Izard, 1960), and social-cognitive and communication abilities (Burleson, 1994). It is a matter of perception, because as long as the children perceive a level of similarity the similarity-attraction hypothesis holds (Montoya et al., 2008; Selfhout et al., 2009).

In this article we focus on two aspects of similarity. The first is to create a sense of shared interests between the child and the robot. Having shared interests is one of the key factors, together with self-disclosure, necessary to experience a friendship (Youniss, 1982; Hartup, 1989; Doll, 1996; Parks and Floyd, 1996; Lang and Fingerman, 2003; Chatterjee, 2005). By disclosing things that match the children’s interest the robot can possible create a sense of shared interests.

The second aspect of similarity we focus on is to match the robot’s behaviors to the personality of the child. Personality is an important factor that influences whether two individuals “hit it off” or feel “no connection” (Svennevig, 2000). In particular whether their extraversion trait matches determines how much they self-disclose to each other (Cuperman and Ickes, 2009). We need an explicit robot behavioral design that matches the extraversion level of children.

### 2.4 Robot behavior design for extraversion

Within the field of human-robot interaction attempts of personality matching is often used to influence various facets of the interaction (Robert, 2018). A strategy to further support self-disclosure is to match the behavior profile of the robot with the extraversion level of the child. Previous attempts of adapting robot behavior to the extraversion level of the child have gained mixed results (Robert, 2018). For example, extraversion matching seems to be effective for motivating people to do exercises (Tapus and Mataric, 2008) or repetitive tasks (Andrist et al., 2015). However, in a quiz game with a robot advisor it did not matter if the extraversion level of the robot and the player matched (Mileounis et al., 2015).

Designing specific introvert and extravert robot behaviors is not trivial. For example, children could not distinguish between introvert and extravert robot behaviors in a mimicking game (Robben et al., 2011). However, if participants perceive a difference in extraversion they prefer the robot that matches them (Aly and Tapus, 2013). A lot of different aspects of the robot’s behavior can be manipulated to create a profile aimed at introverted and extraverted children. For example, the language it uses or the movements it makes (Aly and Tapus, 2013; Craenen et al., 2018). We opted for an ensemble strategy where multiple modalities are manipulated along one axis, arousal, to create two distinct behavior profiles. The robot has a high and low arousal behavior profile to match the behavior typically displayed by extraverted and introverted children respectively (Mairesse et al., 2007). In Section 3.4 the behavior profiles are discussed in more detail.

### 2.5 Child-robot relationship formation

In recent years more research has been conducted to understand if and how children form relationships with social robots (van Straten et al., 2020). A clear pattern is that children not only consider the robot as an agent capable of relationships (Beran and Ramirez-Serrano, 2011), they attribute mental states to robots (Kahn et al., 2013; Desideri et al., 2020; Di Dio et al., 2020) allowing robots to be perceived as an intentional agents that want to engage in a relationship. Not unimportant, research also showed that children want to form relationships with robots (Cowley and Kanda, 2005).

In this article we look through the lens of the getting acquainted interaction and its effect on relationship formation. An important indicator for the successful start of a relationship is an increase of positive affect (McIntyre et al., 1991; Baumeister and Leary, 1995). Vittengl and Holt (2000) found that the perceived amount of self-disclosure and the social attraction between partners predict the increase of positive affect. Meaning that self-disclosure facilitation is not only important for getting acquainted, but plays a direct role in relationship formation. In the second user study we replicate the work of Vittengl and Holt (2000) with child-robot dyads to explore the importance of self-disclosure for child-robot relationship formation.

## 3 Design specifications for child-robot self-disclosure elicitation

We developed four different robot behavior components. They all aim to contribute to support children to comfortably self-disclose to the robot. We adopt the concept of interaction design patterns (IDPs) as the format for the design specifications. Design patterns originate from architecture where Christopher Alexander observed countless patterns in buildings and towns and described them systematically in order for others to use them when constructing new or improving existing structures. Alexander (1977) stated that “each pattern describes a problem which occurs over and over again in our environment and then describes the core of the solution to that problem, in such a way that you can use this solution a million times over, without ever doing it the same way twice”.

Design patterns found its way to software engineering in the 90s (Gamma et al., 1995) and human-computer interface design in the early 2000s (Tidwell, 1999). It lead to the creation of pattern libraries that designers still benefit from (e.g., (Souce Making, 2021) or (Toxboe, 2021)). Kahn et al. (2008) introduced interaction design patterns for human-robot interaction, but unfortunately it has not caught on yet (with exceptions, for example, by Sauppé and Mutlu (2014) and Neerincx et al. (2016)). If done right interaction design patterns specify concrete robot behaviors and form blueprints for other researchers to replicate and re-use the designs. An essential feat to increase the replicability of HRI studies, which is increasingly becoming more important (Irfan et al., 2018; Belpaeme, 2020; Hoffman and Zhao, 2020).

Kahn et al. (2008) argue that their patterns are meant as a starting point and are not yet specific enough nor validated to fully function as a true pattern should. In this paper we start with Kahn et al. (2008)’s first pattern *The Initial Introduction* and specify more concrete robot behaviors and subsequently validate them with two in-the-wild user studies.

The first robot behavior component is a set of five conversational design patterns to structure the getting acquainted conversation. The second component is a tutorial getting children up to speed on how to talk to the robot. The third component is a reciprocation strategy that guides the robot’s responses to children self-disclosures. There are two versions which we will compare in user study 1. The fourth and final component are a pair of behavioral profiles manipulating the arousal level of the robot. A high arousal profile for extraverted children and a low arousal profile for introverted children. These profiles will be evaluated in user study 2, together with the design patterns and the tutorial.

### 3.1 Conversational design patterns - robustly eliciting self-disclosures I

In order to get acquainted with the child, the robot needs to autonomously elicit and process the child’s self-disclosures. The most effective way to do this is by asking closed questions that require one-word answers (Junqua and Haton, 2012). However, this would result in an interrogation rather than a getting acquainted conversation, possibly negatively impacting the willingness of children to self-disclose (Weizenbaum, 1966). To deal with this problem we developed a five conversational design patterns that need to provide enough structure for the robot to effectively process self-disclosures, while being stimulating for self-disclosure elicitation.

Interaction design patterns provide a blueprint for other researchers and developers to replicate and re-use the designs. Ever since the replication crisis in psychology, a field interwoven with the field of human-robot interaction, safeguarding the replicability of HRI studies has become more important (Irfan et al., 2018; Belpaeme, 2020; Hoffman and Zhao, 2020). The patterns we put forward each contain a description of the *problem* they try to solve, the core *principles* of the solution, and a specification and example of the *solution* (i.e. how we implemented it).

#### 3.1.1 IDP1: Pairing closed and open questions

**Problem.** When the robot only asks closed questions to elicit self-disclosure it affects the kind of relationship the robot has with the child. It shifts towards a power relationship, rather than a friendship, where the child has less autonomy over what they can disclose. This not only limits the amount and intimacy of self-disclosure, but also inhibits relationship formation (Derlega and Chaikin, 1977).

**Principle.** In an ideal situation the children can freely respond and even ask questions in return. Unfortunately, the technical limitations of speech recognition and natural-language understanding for children currently prevent this from being realized (Belpaeme et al., 2013a; Kennedy et al., 2017). However, it is possible to use speech activity detection to detect when children are talking. This opens up the possibility to ask open questions, that the robot does not (need to) process, allowing children to freely respond. This would return some of the autonomy back the children.

**Solution.** This design pattern introduces two types of questions, closed and open. Closed questions require a specific valid answer and present those answers in the phrasing of the question. A valid answer is an answer that can be recognized and processed by the robot. A (set of) valid answer(s) always needs to be prespecified. Closed questions are either “yes/no” or multiple choice questions. Open questions have no valid answer, i.e. accept all answers. The robot will only wait for the child to finish answering and will not process the answer.

The closed and open questions always come in pairs. The robot first asks a closed question. For example, “Do you like chocolate, yes or no?“. Using the answer of the child, the robot asks a open follow-up question. For example, “What is the best kind of chocolate and why is it your favorite?“. The closed questions provide all the information the robot needs to personalize future interactions, while the open questions allow the children to freely respond increasing their autonomy.

#### 3.1.2 IDP2: Pseudo-open questions

**Problem.** A pitfall of overly structured dialog scripts is that over time the pattern of interchanging closed and open questions might get dull, resulting in children losing interest.

**Principle.** Adding more variation to the interaction is one way to increase long-term engagement (Leite et al., 2013). Specifically, adding another type of question that can process self-disclosures would be helpful. By carefully designing a question (Boynton and Greenhalgh, 2004) and by knowing the interests of children it should be possible to accurately predict their answer. This opens-up the possibility to ask a pseudo-open question.

**Solution.** A pseudo-open question requires a valid answer but the possible answers are not included in the question. This might give the illusion that any answer is possible, increasing the autonomy of children (Derlega and Chaikin, 1977). However, by carefully choosing the topic, phrasing it to elicit a short a specific response, and if necessary by doing a pilot study, most possible answers can be predicted. For example, “What is your favorite food?“, might be a too broad question with a high risk of missing an answer, whereas for a more specific question like “What is your favorite desert?” it is easier to get a (mostly) complete list of expected answers (to accommodate the speech recognition system).

#### 3.1.3 IDP3: Positive backchanneling

**Problem.** Self-disclosure elicitation is not only about asking questions, but also about responding appropriately to those disclosures (Berg, 1987).

**Principle.** At the bare minimum the robot must acknowledge a response by the child. Better yet, the robot responds to what is being said (Berg, 1987). This is what backchanneling is for (Park et al., 2017).

**Solution.** The robot uses three different backchannel responses: non-lexical, phrasal, and substantive (Young and Lee, 2004). A non-lexical backchannel is a vocalized sound aimed to show the child that the robot is actually listening. For example, “uhuh”. A phrasal backchannel is a short verbal response to acknowledge the answer of the child. For example, “That’s my favorite too!“. A substantive backchannel is aimed to elicit an extended answer by the child. For example, “Go on. Tell me more”. This last one is especially suitable for open questions.

#### 3.1.4 IDP4: Touch-based recognition and repair pipeline

**Problem.** Speech interaction is an important aspect for child-robot bonding (Belpaeme et al., 2013b). But given the overall poor performance of speech recognition for children (Kennedy et al., 2017), a robust repair mechanism needs to be in place.

**Principle.** Instead of only relying on speech we can make use other input modalities of the robot (Belpaeme et al., 2013b). Touch has been found to have a positive impact on self-disclosure elicitation by robots (Hieida et al., 2014; Shiomi et al., 2017). The robot has various buttons that can be used for a touch-based repair mechanism.

**FIGURE 1:**
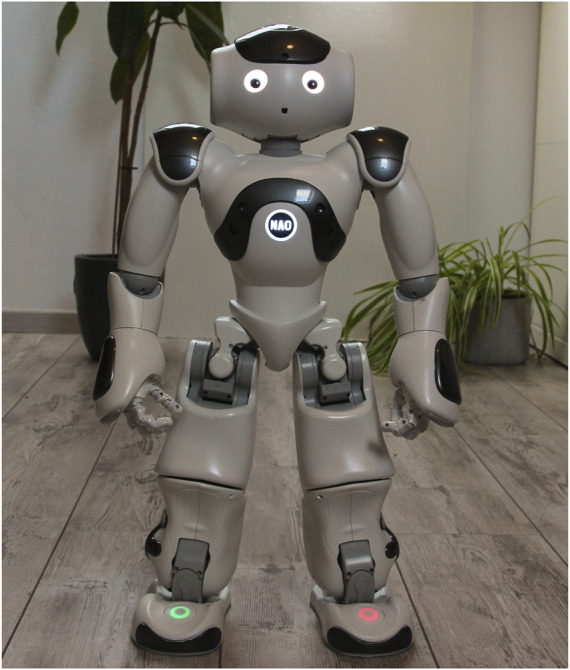
The bumper (button) on the robot’s foot below the green light means yes and the bumper below the red light means no.

**Solution.** To not discourage talking to the robot we allow for two speech recognition attempts per question. If after two attempts no response was successfully recognized, the robot switches to the touch modality. On its feet the Nao has a ‘bumper’ that can be pressed. One foot is the yes-bumper (signalled by a green light) and the other a no-bumper (signalled by a red light). See Figure 1. For “yes/no” questions the appropriate bumper can be pressed directly. For multiple choice and pseudo-open questions the robot lists a number of popular answers and instructs the child to press the yes-bumper when the robot calls out the right answer. Two touch attempts are allowed. In case that would fail, the robot moves on to the next question. Note that this pattern does not repair incorrectly recognized speech.

#### 3.1.5 IDP5: Six-step turn-taking

**Problem.** A child-robot conversation is difficult for both child and robot at first. Instructions help the child. But even little misconceptions can complicate things. For example, even though given the opportunity to answer freely to open questions, some children may still answer verbosely to closed questions. The robot has trouble processing these answers.

**Principle.** By consistently and appropriately directing the turn-taking, children should quickly pick-up how to smoothly talk to the robot, while the robot is provided with a robust structure for asking various questions and providing appropriate responses.

**Solution.** A repeating six-step turn-taking mechanism. The steps: 1) the robot takes the initiative by starting off with a closed or pseudo-open question, 2) followed by an answer from the child, 3) which in turn causes the robot to respond. 4) The robot subsequently asks the child to explain their answer, 5) followed by a response by the child, 6) that the robot acknowledges with a response. A response by the robot can either be a backchannel or a reciprocal self-disclosure by the robot (see Section 3.3). This composite pattern builds on patterns IDP1 to 4.

### 3.2 How to talk to Me tutorial - robustly eliciting self-disclosures II

Unlike meeting new people (mostly), the first step of meeting a new robot is always figuring out how to talk to it. Although we make a considerable effort to design a getting acquainted conversation that is easy to participate in for children, countering the technological constraints with straightforward dialog management solutions, it is not enough to guarantee a smooth interaction from the start. The lack of experience and expectations based on science fiction stories and the child’s imagination requires a robust tutorial on how to communicate with the robot (Ligthart et al., 2017).

A good tutorial not only helps the child to figure out how to talk to the robot, it also helps to manage their expectations of the robot. In earlier work we found that if children expect too much from the robot they get so disappointed they disengage (Ligthart et al., 2017). Aligning expectations with the true capabilities of the robot is an essential step in the child getting acquainted with the robot. Partly, this can be done by properly introducing the robot and explaining what it can and cannot do. We argue that it is important, and perhaps more fun, to make the robot responsible for expectation management.

We did that in the following ways. Firstly, the robot is the one who instructs the child how to talk to it. This includes explaining that the robot is the one who asks the questions and to answer children have to speak loud and clear and wait for the beep. The robot will beep and its ears will light up to indicate it is listening. Secondly, the robot asks two practice questions. One that the child can answer verbally (i.e. the default way) and the other using the touch-based repair mechanism. Thirdly, we included two activities to get familiar with the robot’s capabilities. We designed a tickle game that allows the children to touch various buttons and sensors of the robot, making it laugh when they do. The second activity was the robot showing off its dance moves.

Finally, we created a narrative around the robot to manage the children’s expectations further. The narrative includes a motivation for why the robot is at school talking the children. The robot is it at school to practice for it is upcoming internship as care robot in the hospital. The robot further tells the children it is very curious about them, because it feels they are the most similar to it. The narrative of practicing is meant to include the (speech recognition) errors and other mishaps, that are abound the happen, in the narrative. Hopefully children experience those errors as less disruptive. The robot’s strong interest in children aims to explain why it is the one asking most of the questions. And the robot’s feeling of similarity is indented to prime the children to be more aware of similarities.

### 3.3 Reciprocation strategies - responding to self-disclosures

Not only the structure of the conversation, but also its content is important for getting acquainted and eliciting self-disclosure. We use the term *robot repertoire* to describe the collection of questions, backchannel responses, and robot-disclosures the robot can share.

The questions are mostly about identifying the favorite item for a particular topic. For example, the favorite pet or favorite holiday destination. The topics included in our implementation are sports, leisure activities, books, pets, seasons, colors, holidays, and television. These topics are selected because they aim to directly elicit self-disclosures of an appropriate intimacy level for a getting acquainted interaction (Altman and Taylor, 1973). Moreover, children are used to these kind of questions. For example, they are often included in friendship books.

The robot can respond in two different ways. It either gives a backchannel response or it reciprocates the child’s self-disclosure by giving a robot-disclosure. Robot-disclosure is important due to the reciprocal nature of self-disclosure (Birnbaum et al., 2016; Burger et al., 2017). Robot-disclosures are fictional anecdotes of the robot’s personal life. Children are generally aware that these anecdotes are part of a story, but have the tendency to play along for the sake of the story (Belpaeme et al., 2013a).

The following example, of an actual conversation between child (C) and robot (R), shows the interaction design patterns for a getting acquainted conversation instantiated with robot repertoire elements.

R: “What is your favorite pet?” [pseudo-open-question]
C: “A dog.’ [recognized by speech recognition]
R: “Oh nice, why is a [dog] your favorite pet?” [open-question]
C: “Because they are very playful.” [speech activity detection]
R: “Go on. Tell me more.” [substantive backchannel]
C: “We have a Golden Retriever at home.”
R: “Cool! Let me tell you about a dog I know. He’s called Buddy. Buddy really likes to play fetch. One time he even jumped in the river to get his ball back. Silly dog.” [robot-disclosure]

One of the questions addressed in this article is how to design a reciprocation strategy that further reinforces self-disclosure. A straightforward strategy is to explicitly like what the child likes and offer a robot disclosure about that topic. This strategy could increase the feeling of similarity between the child and the robot. The more the child feels similar to the robot, the more it supports relationship formation and self-disclosure (Selfhout et al., 2009). However, always explicitly liking what the child likes might get boring after a while. More variation in the responses of the robot is important for keeping them interested (Leite et al., 2013). A robot that has its own character and preferences might be more appealing (Luria, 2018). The robot could acknowledge the answer of the child, but express it likes something else. For example if the child responds it likes dogs the robot could respond with “Dogs are great. I like bunnies, because they are super fluffy”.

In the first user study, discussed in Section 4, two reciprocation strategies are compared. The first is the strategy where the robot explicitly likes what the child likes (explicit strategy). The other is a more nuanced strategy, based on giving the robot preferences of its own. In four out of five responses the robot matches the answer of the child, but just gives a robot disclosure about that answer and does not explicitly state that the answer is its favorite as well. Every five responses the robot states it likes a different answer and gives a robot disclosure about that answer (nuanced strategy).

### 3.4 Extraversion adaption - increasing self-disclosure

To further increase our efforts to elicit self-disclosure we have designed two behavior profiles for the robot. One profile is specifically designed for extraverted children and the other for introverted children. We looked at a wide range of typical marker differences for introvert and extravert human behavior. We translated these behavioral differences to a number of behavior settings for the robot (see Table 1).

**TABLE 1:** Behavior settings for the two arousal-based behavior profiles.

**Behavior setting**	**High arousal**	**Low arousal**
Speech speed	100%	90%
Speech volume	49	40.5
Language style	directive	interrogative
Emotion words	strong	weak
Speech activity detection interval	2–3s (100%)	2.5–3.75s (125%)
Gestures amplitude	100%	60%
Gestures speed	100%	50%
Head movement speed	100%	75%
Breathing animation	20 bpm	10 bpm
Activity order	Dance - game	Game - dance

Extraverts have a higher arousal level than introverts. For example, extraverts talk more, faster, louder, use fewer pauses, and less formal language, produce responses with shorter latency, use more positive emotion words, and agree and compliment more (Mairesse et al., 2007). To create matching robot behaviors we designed a behavior profile with a higher arousal level for extraverts and a behavior profile with a lower arousal level for introverts. The low arousal robot talks slower and softer compared to high arousal robot. It also waits longer for a response by the children.

The low arousal robot uses more tentative words and uses less social and weaker positive emotion words. For example, “Cool. Could you tell me more?”. The language of the high arousal robot, on the other hand, is more directive (i.e., less tentative words) and contains more social and stronger positive emotions words. For example, “That is an awesome choice! Tell me more”.

Furthermore, as in (Aly and Tapus, 2013; Craenen et al., 2018), we have also varied the amplitude and speed of the movements. We varied the arms, head, and torso separately. The arms display random gestures when the robot is talking. While keeping the frequency of the gestures the same they are slower and smaller for the low arousal robot. The robot nods its head while listening. The head movements of the low arousal robot are slower, reducing the frequency. Finally, the torso moves slowly from left to right to simulate breathing. The low arousal robot has less ‘breaths’ per minute (bpm).

The arousal level of the robot is not only dependent on it is behavior profile, but also on the type of activities one does with the robot. To address this aspect, two activities were included in the getting acquainted interaction. A high arousal dance and a low arousal tickle game. The thirty second classic Gangnam Style dance animation and music is used for the dance activity. During the thirty second tickle game the children are invited to touch certain buttons of the robot to make it laugh (default Nao laughing animations are used).

In the second user study, discussed in Section 5, the effect of a high and low arousal robot on children’s self-disclosure behavior is measured. The getting acquainted conversation is the central part of the interaction. For the high arousal robot, the dance activity was included before the conversation and the tickle game after. For the low arousal robot a reverse order was used.

The exact settings as listed in Table 1 were established via rapid prototyping and small pilots with children and adults. The settings are meant to create enough contrast between both behavior profiles to match with introverted and extraverted children, while still resulting in a decent conversational partner. For example, if the robot would talk too slow or too fast children would not be able to understand the robot anymore.

## 4 User study 1 - reciprocation strategy

The first user study of this article is centered around comparing the explicit and nuanced reciprocation strategies. The child and the robot have a conversation where the robot asks the child about their favorite things. The robot reciprocates by sharing its favorite things. The robot either always matches the child’s answer and states that it is their favorite as well (explicit strategy). Or the robot matches the child’s answer in 4/5 of the cases without explicitly stating it is their favorite and in 1/5 cases the robot has a different favorite thing. This study furthermore served as a pilot that helped further shape the conversational patterns and the tutorial.

### 4.1 Research question and hypotheses

The aim of the reciprocation strategy is to elicit children to self-disclosure and to create a sense of similarity, while remaining authentic and creating a joyful conversation. The first research question is

RQ1.1 Which reciprocation strategy (explicit vs. nuanced) a) elicits the most self-disclosures and creates a stronger sense of b) similarity, c) authenticity, and d) enjoyment?

Because in most of the cases (4/5) in the nuanced strategy, and in all explicit responses, the robot disclosures an anecdote about the child’s answer, we expect no difference in children’s perceived similarity (*H*1.1_*b*_). However, because the nuanced robot is less explicit and mixes it up with it is own preferences (1/5 cases), we expect children to perceive the robot as more authentic (*H*1.1_*c*_) and enjoy the conversation more (1.1_*d*_). And because it shows more variety in responses we expect children to self-disclose more to a robot using the nuanced reciprocation strategy (*H*1.1_*a*_).

### 4.2 Methods

#### 4.2.1 Participants

30 children (age 8–10, average age 9 years. o., 20 boys and 10 girls) completed the experiment. The children were all part of the same class in school and were recruited by their teacher upon our request. Their parents received an information brief and consent form to sign in advance. This study was approved by the Human Research Ethics Committee (HREC) of the Delft University of Technology. Children with the same age and gender were randomly paired. The pairs were randomly split and assigned to one of the two conditions (*N*_exp_ = 15, *N*_*nua*_ = 15).

#### 4.2.2 Experimental design

We ran a between-subjects study with the reciprocation strategy (explicit versus nuanced) as the single independent variable. There were four dependent variables: the amount of self-disclosure, perceived similarity, authenticity, and enjoyment.

#### 4.2.3 Measures and instruments

##### 4.2.3.1 Amount of self-disclosure

The video recordings were used to transcribe the conversations. Each answer to a question was considered a valid self-disclosure opportunity. Two annotators counted the number of individual statements during each self-disclosure opportunity. Summing these counts resulted in the total amount of self-disclosure per participant. To summarize the instruction set for the annotators, every part of the response that is or could syntactically be separated by either a comma or an ‘and’ should be counted as a unique statement. For example, “I always wanted to have a cat” counts as one and “I like to play football and tennis” counts as two. An exception however is when two parts of a statement belong to the same concept. For example, “My favorite TV-show is Tom and Jerry” counts as one. Differences in counts between annotators were resolved via discussion.

##### 4.2.3.2 Similarity, authenticity, and enjoyment

Participants were asked to rate their similarity to the robot, the authenticity of the robot, and their enjoyment of the conversation with two items each on a 5-point rating scale (totally disagree 1) - totally agree (1)). Items were averaged to get the test scores for each measure.

#### 4.2.4 Materials and set-up

The experiment took place in a separate room in the school. An orange V4 Nao robot was used in a wizard of Oz (WOz) set-up. A graphical WOz interface was used to navigate the conversation. The wizard could manage the turn taking and select the appropriate response based on the child’s answer and the reciprocation strategy with a simple button click. Because there was only one room available for the study, the wizard was present in the room. The wizard was introduced as a student observant and was seated behind the participant to keep the illusion of an autonomous robot alive. None of the participants communicated with or paid attention to the wizard. The interaction was recorded on video with audio using a A Sony HDR-handycam.

#### 4.2.5 Procedure

Participants came to the experiment room one-by-one to interact with the robot. The robot would ask questions in the form of “what is your favorite …” about the following topics: pets, sports, colors, school subject, and holiday destination. After each answer the robot would give a short anecdote about its favorite answer. The explicit robot would literally say “that is my favorite too” in every response. The nuanced robot matched the answer of the child but just gave the anecdote about that answer. In one out of five responses the nuanced robot would explicitly express it liked something else and give an anecdote about that instead. After the interaction participants were asked to fill in a questionnaire. This all took 20 min per child, 10 min for the interaction and 10 for filling in the questionnaire.

### 4.3 Results

**FIGURE 2:**
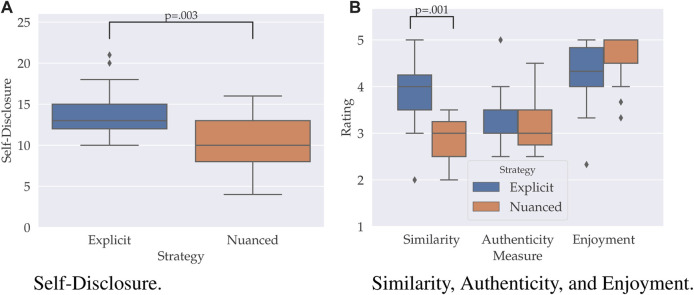
Boxplots showing the distribution of the amount of self-disclosure **(A)** and self-report ratings **(B)** for the robot using an nuanced and explicit reciprocation strategy respectively.

Mann-Whitney U test’s were run to determine if there were differences in self-disclosure, similarity, authenticity, and enjoyment scores between a robot using the explicit or nuanced strategy (see Figure 2). A Bonferroni correction was applied to account for multiple testing (*α* < 0.0125). Distributions of the self-disclosure scores and for each rating for both strategies were similar, as assessed by visual inspection. Data are median [quartiles]. Participants statistically significantly self-disclosed more to the explicit robot (13 [12, 15]) than the nuanced robot (10 [8, 13]), *U* = 43.5, *z* = −2.9, *p* = 0.003, *η* ^2^ = 0.27. Participants furthermore felt statistically significantly more similar as the explicit robot (4 [3.5, 4.5]) than the nuanced robot (3 [2.5, 3.5]), *U* = 36.5, *z* = −3.2, *p* = 0.001, *η* ^2^ = 0.33. No significant difference between the explicit and nuanced strategies were found for the feeling of authenticity (3 [2.5, 4] vs. 3 [3, 3.5]) and enjoyment (4.7 [4, 5] vs. 4.7 [4, 5]), *U*′*s* > 101, *z*′*s* > − 0.5 and *p*′*s* > 0.653.

## 5 User study 2 - A getting acquainted conversation

With the second user study we set out to thoroughly evaluate the getting acquainted conversation as a whole and zoom in on the specific design components. We aimed to validate the conversational interaction design patterns, test the effect of matching the robot’s arousal level on self-disclosure, explore the relationship between children’s sense of similarity on how well children got acquainted with the robot, and explore the impact of the tutorial on how well children learned to talk to the robot. Finally, we replicated the study of Vittengl and Holt (2000) to explore whether child-robot relationship formation follows a similar process as child-child relationship formation.

### 5.1 Research questions and hypotheses

User study 2 boils down to the following three central questions.

*How effective are the design components for facilitating a getting acquainted conversation that enables*

1) the robot to get acquainted with the child?
2) the child to get acquainted with the robot?
3) child-robot relationship formation?

The effectiveness and robustness of the conversational design patterns to elicit self-disclosure is key for the robot to get acquainted with the child. The following sub-questions are formulated to investigate the individual patterns.

*RQ*2.1*a*. How successful are the different questions in eliciting self-disclosure? Indicated by the response rates of the three types (closed, pseudo-open, and open) of questions. *[IDP1, 2]*
*RQ*2.1*b*. Do children give valid (i.e. predicted) answers to the pseudo-open and closed questions? *[IDP2, 1]*
*RQ*2.1*c*. How successful are the backchannels for eliciting self-disclosure? Indicated by the response rates to the three types (non-lexical, phrasal, and substantive) of backchannels. *[IDP3]*
*RQ*2.1*d*. How successful is the recognition and repair pipeline and is the touch-based mechanism an effective alternative? Indicated by the recognition performance. *[IDP4]*
*RQ*2.1*e*. How often does speech recognition fail and what are the causes? *[IDP4]*
*RQ*2.1*f*. How often is an answer incorrectly recognized and how do children respond to those mistakes? Due to the lack of a repair mechanism for incorrectly recognized speech it is important to assess the impact of those mistakes. *[IDP4]*
*RQ*2.1*g*. How do speech recognition errors influence the interaction? *[IDP4]*
*RQ*2.1*h*. How successful is the six-step turn-taking mechanism? Success means that children give a concise answer to the initial closed/pseudo-open question and leave a verbose answer for the follow-up open question. *[IDP5]*

The arousal behavioral profiles are designed to stimulate self-disclosure elicitation when they match with the children’s extraversion trait. To study whether a matching effect occurs we formulated the following research questions. Note that we measured positive affect before and after the interaction and used the change in positive affect as a measure for the success of the relationship formation.

*RQ*2.2 What is the effect of the arousal level of the robot on self-disclosure for introverted and extraverted children respectively?
*RQ*2.3 What is the effect of the arousal level of the robot on positive affect change for introverted and extraverted children respectively?

Following the theory that matching levels of extraversion lead to a better interaction and more self-disclosure (Byrne et al., 1967; Cuperman and Ickes, 2009) we hypothesize that extraverts will self-disclose more (*H*2.2*a*) and more intimate (*H*2.2*b*) to a high arousal robot and that introverts self-disclose more (*H*2.2*c*) and more intimate (*H*2.2*d*ays) to a low arousal robot. We expect the same results (*H*2.3*a* − *d*) for positive affect increase (i.e. indicator for relationship formation). We furthermore expect that, just like within human-human dyads (Cuperman and Ickes, 2009), extraverts are more willing to self-disclose than introverts (*H*2.2*e*).

The tutorial’s main objective is to teach children how to comfortably talk to the robot. Knowing how to talk to the robot is also an important part of getting acquainted with the robot. We formulated the following question.

*RQ*2.4 What is the contribution of the tutorial to children getting acquainted with the robot?

In user study 1 we demonstrated that the explicit reciprocation strategy not only elicits more self-disclosure, it also creates a sense of similarity between children. That is why we selected the explicit reciprocation strategy as the default. We are interested in exploring the impact of a stronger sense of similarity on the getting acquainted interaction. We have the following research questions.

*RQ*2.5 What is the relationship between the feeling of similarity and a) self-disclosure and b) positive affect change?

Experiencing similarity is a facilitating factor when children getting acquainted and form relationships amongst each other (Hartup, 1989; Doll, 1996; Selfhout et al., 2009). We therefore expect that there is a positive relationship between children who feel more similar to the robot and how much they self-disclose (*H*2.5*a*) and the change in positive affect (*H*2.5*b*).

Finally, to evaluate the effectiveness of the interaction design patterns to enable relationship formation between the child and the robot we replicated the study by Vittengl and Holt (2000). They studied relationship formation between dyads of young adults during a short getting acquainted interaction. We replicated their set-up to study child-robot relationship formation.

*RQ*2.6*a* What is the effect of the getting acquainted interaction on positive affect?
*RQ*2.6*b* What is the relationship between self-disclosure and social attraction and positive affect change?

Similarly to the original study we expect that children experience more positive affect after than before the interaction (*H*2.6*a*) and that the positive affect increase is predicted by the perceived amount of self-disclosure and social attractiveness of the robot (*H*2.6*b*).

### 5.2 Methods

#### 5.2.1 Participants

75 children, between 8 and 11 years old, of two Dutch primary schools (school A and B) completed the experiment. 45 girls and 30 boys were recruited from two classes per school. In school A and B respectively 41 and 34 children participated.

The age, gender, and extraversion level (extravert (E) or introvert (I)) of participants were kept balanced while assigning participants to a condition. Per school participants with the same age, gender, and extraversion level were randomly paired. Randomly one was assigned to the matching (+) robot and the other to the mismatching (−) robot (*N*_*E*+_ = 18, *N*_*E*−_ = 19, *N*_*I*+_ = 18, *N*_*I*−_ = 20).

Participants were recruited by their respective teachers upon our request. Their parents received an information brief and consent form to sign in advance. This study was approved by the Human Research Ethics Committee (HREC) of the Delft University of Technology.

#### 5.2.2 Experiment design

We used a 2 × 2 between-subjects study design to research the effect of (mis)matching the arousal behavioral profile of the robot with the extraversion level of the children on self-disclosure (*RQ*2.2) and relationship formation (*RQ*2.3). The two independent variables are the extraversion of the child (introvert versus extrovert) and the behavior profile of the robot (high or low arousal). The three dependent variables are the amount and the intimacy of self-disclosure and positive affect change. The interaction design patterns, reciprocation strategy, and tutorial were implemented across all conditions. All the interactions that were included in the experiment were used to evaluate the IDPs (*RQ*2.1), influence of similarity (*RQ*2.5), and the contribution of the tutorial (*RQ*2.4). Likewise all dyads were included in the replication of Vittengl and Holt (2000)’s study (*RQ*2.6).

#### 5.2.3 Measures and instruments

##### 5.2.3.1 Interaction design pattern effectiveness

Using the video recordings, all conversations between participant and robot were transcribed to text. Using the transcriptions we determined for each question (*RQ*2.1*a*) and backchannel (*RQ*2.1*c*) attempt whether it elicited a response by the participant and whether it was valid (*RQ*2.1*b*). We calculated for each question the average amount of characters and whether it was too verbose (*RQ*2.1*h*).

We logged each speech recognition and, in case of a failure, repair attempt. This allowed us to calculate the success rate of each step in the recognition and repair pipeline (*RQ*2.1*d*). Annotators annotated the cause for each failure (*RQ*2.1*e*). We also logged every time the speech recognition recognized an answer incorrectly together with the observed response to that error by the participant (*RQ*2.1*f*). Finally, a correlation analysis with the total amount of speech recognition errors per participants and relevant outcome measures can be done to explore what influence those errors might have on the interaction (*RQ*2.1*g*).

##### 5.2.3.2 Participant and robot extraversion

To categorize participants either as introvert or extravert we used the extraversion subscale of the Hierarchical Personality Inventory for Children (HiPIC) (Mervielde and De Fruyt, 1999). Teachers rated for each participants the 32 items from the extraversion subscale. We used a mean split, per classroom, to label participants as introverts or extraverts. We selected 8 suitable items from the HiPIC questionnaire and asked the participants to rate the extraversion level of the robot.

##### 5.2.3.3 Self-disclosure

The notion of self-disclosure is a multilayered concept. We measured two different aspects: the amount and the intimacy of self-disclosure. Two annotators used a set of instructions to annotate the responses. Annotator disagreements were resolved in a discussion after completing the annotations.

The amount of self-disclosure is operationalized as the total count of unique statements related to oneself within all the responses made by a participant. The annotators marked and counted the unique statements per response. Summing these statements resulted in the total amount of self-disclosure per participant. The same annotation instructions were used as in the previous user study.

The intimacy measure of each self-disclosure is based on the Disclosure Intimacy Rating Scale for child-agent interaction. The scale contains four increasing levels of intimacy that are based on the risk of receiving negative appraisal and the perceived impact of betrayal by the listener (Burger et al., 2016). Their research shows that almost all self-disclosures of an initial interaction fall under the first level.

To increase the expressive power of the disclosure intimacy rating scale we designed a level 1 subscale specific to the type of interaction present in our experiment. Using 20 randomly selected statements 5 sublevels were defined. To indicate a relative difference between the levels, a score between 0 and 3 was attached. The levels are related to the type of argumentation given by the participants to justify an answer. In Table 2 the levels are illustrated based on responses to the question “Why is France your favorite holiday destination?“.

**TABLE 2:** Example of self-disclosed statements with an intimacy level and score assigned.

**Level**	**Self-disclosure**	**Score**
No argument	“Because it is my favorite.”	1
Fact	“Because Disneyland is there.”	2
Personal fact	“Because my aunt lives there.”	3
Opinion	“Because it’s the most beautiful country in the world.”	3
Other	“I don’t know” or “What is yours?”	0

The total intimacy score is the summed intimacy scores of each response (not statement). Children can for example have a high amount of self-disclosure but a low intimacy score and vice versa.

##### 5.2.3.4 Tutorial contribution

To investigate what participants remembered from the tutorial we asked them how they would explain how to talk to the robot to a peer. We furthermore made notes about whether children adhered to the instructions that were given during the tutorial.

##### 5.2.3.5 Similarity

We asked participants to rate their perceived similarity to the robot (1 item question) and to motivate their rating.

##### 5.2.3.6 Child-robot relationship formation

For researching relationship formation we have used the same measures for self-disclosure, social attraction, and affect as Vittengl and Holt (2000). The only difference is that we used the Dutch version of the measures. To measure perceived self-disclosure participants were asked to indicate how much they disclosed about 10 different topics. Social attraction is a self-report measure used to capture the social attraction of the participants towards the robot. It is heavily based on the social attraction subscale of the Interpersonal Attraction Scale. The Positive and Negative Affect Schedule (PANAS) was used to measure positive affect before and after the interaction with the robot.

#### 5.2.4 Materials and set-up

An orange V4 Nao robot was used with its default speech recognition software. A Sony HDR-handycam was used to record the interaction on video and audio. All robot commands were executed on the robot by the default Naoqi framework. All custom-made software ran locally on a standard issue Dell laptop[Fn fn2] ^2^.

**FIGURE 3:**
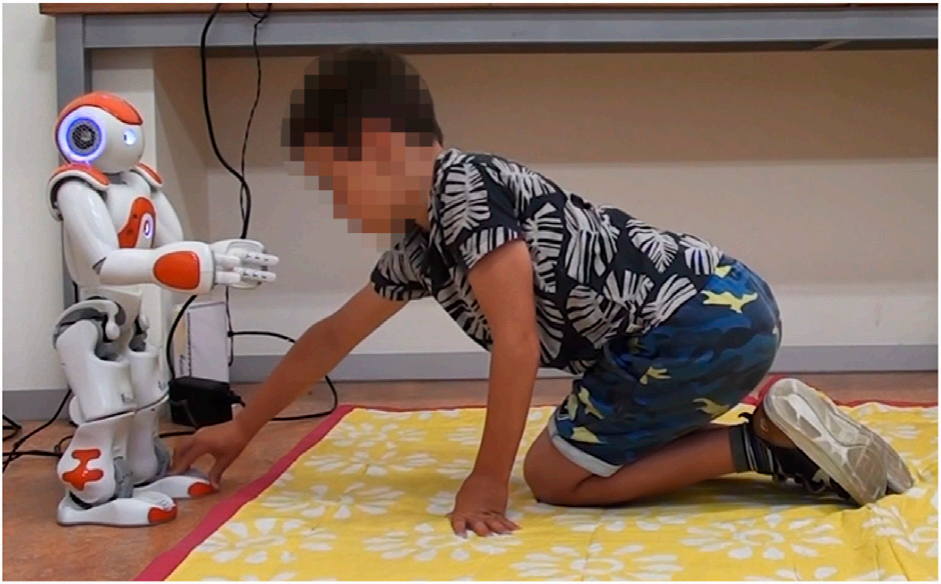
Child pressing one of the answer bumpers on the Nao Robot. The image is a screen shot from the camera.

The experiment took place in two rooms at the school familiar to the participants. One experimenter introduced the participant to the robot and supervised the interaction in one room. And a different experimenter interviewed the children afterwards in the second room without knowing to which condition they were assigned. During the interaction the participants were asked to sit in front of the robot on the floor. The experimenter in the room was seated out of view. The camera was placed on a small stool, to make it blend in without completely obscuring it, perpendicular to the child and robot (see Figure 3).

#### 5.2.5 Procedure

The experiment was a single session study. Data collection took place in a 2 week period. A week before the start teachers filled in extraversion questionnaires for each participant. On the first day both experimenters introduced themselves and the Nao robot, in an idle state, to all participating class rooms. The global procedure was explained and children could ask questions.

Children were collected from the class room one after the other and escorted to the interaction room. Upon entry the robot was hidden from sight. The participants were explained that they would have a conversation with the robot and that afterwards they would be asked to tell us what they thought about it. Furthermore, it was pointed out that they could stop at anytime without consequences or giving a reason.

When the participant was ready the robot was revealed and placed in a squatting position on the ground. The participants were asked to sit in front of the robot on the ground. The experimenter briefly demonstrated where the buttons on the robot were and how to press them. Then the robot and the camera were turned on.

First the robot introduces itself and its purpose. The robot demonstrates and practices with the children how they need to talk to the robot and press its buttons. To showcase the other capabilities of the robot a dance and a tickle game are added to the interaction. One goes before and the other after the getting acquainted conversation. The order is dependent on the behavior profile (see activity order in Table 1). The conversation is the main component. The following topics were included in the conversation sports, leisure activities, books, pets, seasons, colors, holidays, and television. The robot would move from one topic to the next. For each topic the robot would, using conversational patterns, ask the participant questions (see Section 3.3 for an example), typically about their favorite sports, leisure activity, etc. After the conversation the robot would initiate the second activity and after that say goodbye.

After the interaction was over the participants were escorted to the interview room where they were interviewed. The interviewer had no knowledge of the condition of the participant. Finally, the participants were thanked and asked to not discuss the experiment with their peers until the experiment was finished for everyone. The overview of the procedure is below:

1) *Welcome and informed consent check*
2) Introduction
  • Robot introduces itself
  • ‘How to communicate with me’ tutorial
3) Activity 1: dance or tickle game
4) Main getting acquainted conversation
5) Activity 2: tickle game or dance
6) Goodbye
7) *Interview*

### 5.3 Results

#### 5.3.1 Interaction design pattern effectiveness

To answer the evaluation question *RQ*2.1*a* and *RQ*2.1*b* we present the rates of the total and the valid responses to all the questions asked by the robot in Table 3. To answer research question *RQ*2.1*c* we present the response rates to all backchannel attempts in Table 4.

**TABLE 3:** Question response rates and lengths.

**Type**	**#**	**Res. Rate** (%)	**Valid**	**Avg. Chars**
Closed	542	98	97%	9 ± 7
Psuedo-open	285	99	95%	12 ± 10
Open	533	88	n/a	40 ± 32

**TABLE 4:** Backchannel response rates.

**Type**	**#**	**Res. Rate** (%)
Non-lexical	117	21
Phrasal	74	51
Substantive	190	85

**FIGURE 4:**
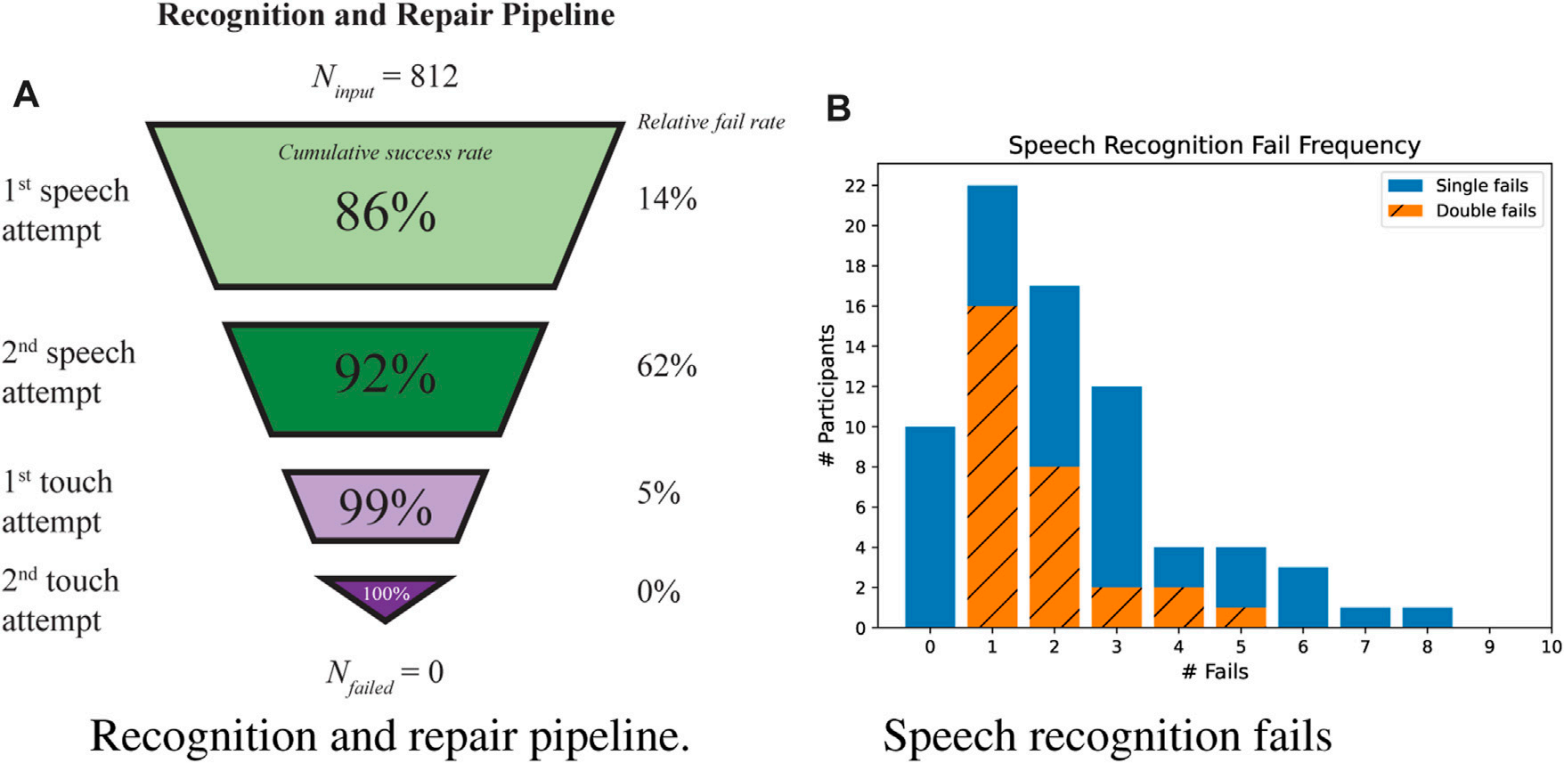
Success rates of recognition and repair pipeline illustrated in a funnel graph **(A)** and frequency graph of how many question a speech recognition error occurs **(B)**.

We evaluated four aspects of the performance of the touch-based recognition and repair pipeline (IDP4). First we looked at the ability to recognize a valid answer (*RQ*2.1*d*). In Figure 4A a funnel overview is presented that depicts the success/failure ratio for all four steps of recognizing a valid answer.

**FIGURE 5:**
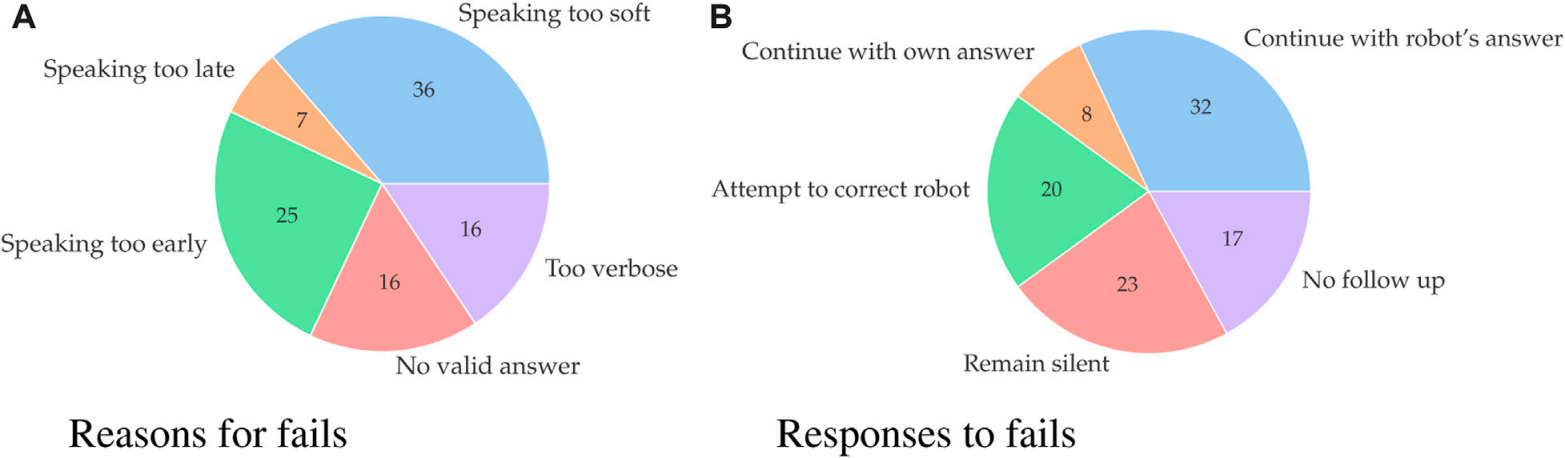
Pie-charts representing reasons for speech recognition fails **(A)** participant’s responses to a follow-up question after their initial answer was incorrectly recognized **(B)**.

The second aspect is speech recognition performance (*RQ*2.1*e*). There were 986 speech recognition attempts. 266 times (27%) the attempt failed. We assigned a reason to each failed attempt. 80% of the fails were due to something the participant did (see Figure 5A). For example, speaking too soft. In the remaining 20% of the cases the participants followed protocol, but the speech recognition failed nonetheless.

The robot asked ten questions. In Figure 4B the frequency of participants who failed with zero or more questions are displayed by the blue non-hatched bars. Each participant had two speech attempts before switching to the touch modality. The orange hatched bars show the frequency of participants that failed two speech attempts on one or multiple occasions. Speech recognition failed on average approximately the same amount of times for participants talking to the high arousal robot (3.6 ± 2.7) and the low arousal robot (3.6 ± 3.2).

The third aspect is the effect of incorrectly recognizing an answer (*RQ*2.1*f*). Of 812 attempts the robot recognized an answer 71 times (8.7%) incorrectly. The different ways participants responded to these recognition errors are displayed in Figure 5B.

The fourth and final aspect is to assess whether there is a relationship between the failed speech recognition attempts (regardless of cause) and the outcome of the interaction a Pearson’s product-moment correlation was run (*RQ*2.1*g*). The included outcome measures are the amount and intimacy of self-disclosures, perceived comfort, social attraction, and positive affect change. The results are shown in Table 5.

**TABLE 5:** Pearson’s correlation results of failed speech recognition attempts with self-disclosure (SD), perceived comfort, social attraction, and positive affect change.

Amount of SD	Intimacy of SD	Perceived comfort	Social attraction	Positive affect
−0.06	−0.30^a^	−0.30^a^	−0.24^b^	−0.08

a Correlation is significant at the 0.01 level (2-tailed).

b Correlation is significant at the 0.05 level (2-tailed).

To answer the evaluation question *RQ*2.1*h* we looked at the average character count for the answers to questions (see final column of Table 3). Of the 812 times a participant responded to a closed and pseudo-open question 28 times (3.5%) they responded too verbosely, resulting in a speech recognition failure.

#### 5.3.2 ‘How to talk to me’ tutorial

The core three instructions of the tutorial were to speak loud and clear and after the beep. 71 (96%) of the children reiterated to speak loud and clear when asked how they would explain peers to talk to the robot. 62 (83%) of the children explicitly mentioned to talk after the beep. It was not annotated, but in general most children displayed self-correcting behavior when speaking too early or too soft. If the child spoke before the beep they would often repeat their answer after the beep. If speech recognition failed the first time children would often speak up and repeat their answer more loudly. A lot of mistakes were prevented by this behavior.

#### 5.3.3 Robot arousal behavior profile

**FIGURE 6:**
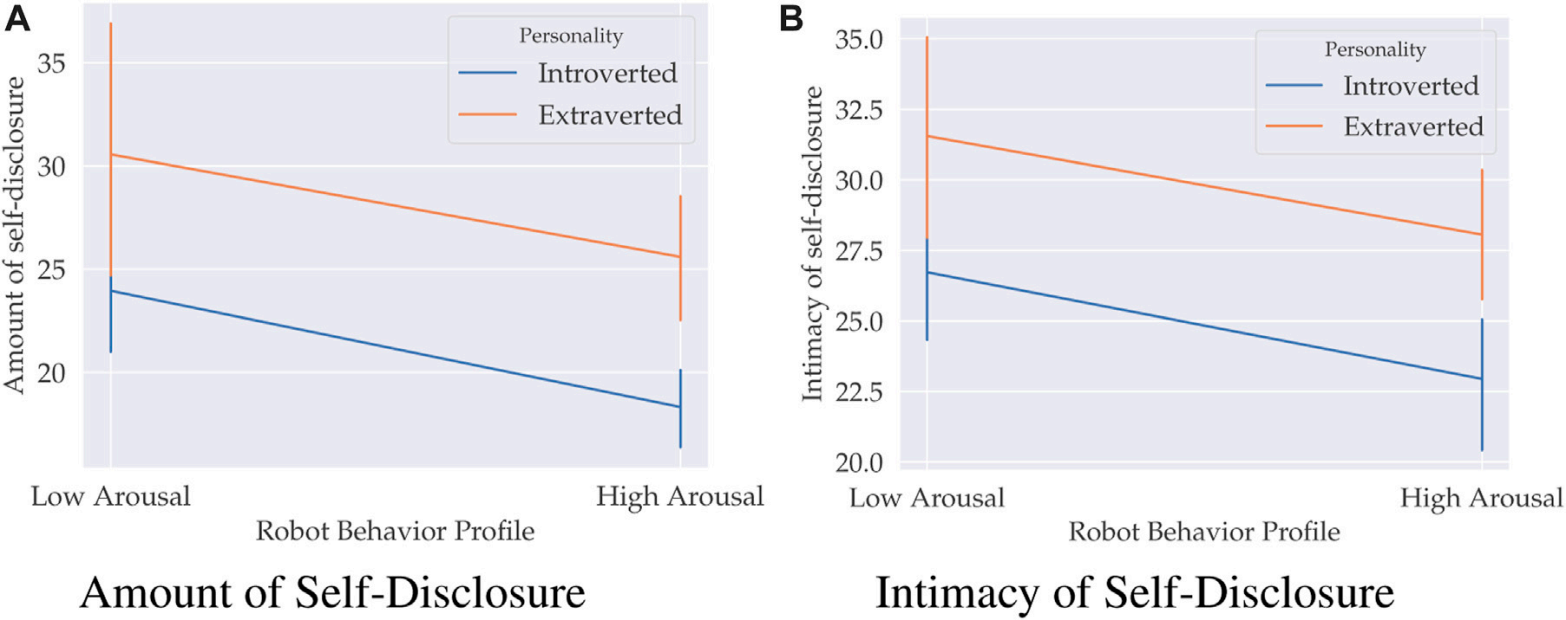
Mean self-disclosure scores (amount in (**(A)** and intimacy in **(B)**) for high and low robot arousal behavior profile and extraverts and introverts with 95% confidence intervals.

A two-way MANOVA was run with two independent variables–participant’s extraversion and the robot’s arousal level–and two dependent variables–the amount and intimacy of self-disclosure (see Figures 6A,B). The interaction effect between extraversion and arousal was not statistically significant, *F* (2, 69) = 0.012, *p* = 0.988, *Pillai’s Trace V* <.001 , *η* ^2^ < 0.001. There was a statistically significant main effect of the arousal on self-disclosure, *F* (2, 69) = 3.501, *p* = 0.036, *Pillai’s Trace V* = 0.092, *η* ^2^ = 0.092. There also was a statistically main effect of extraversion on self-disclosure, *F* (2, 69) = 6.329, *p* = 0.003, *Pillai’s Trace V* = 0.155, *η* ^2^ = 0.155.

An two-way ANOVA was run to examine the effects of participant’s extraversion and the robot’s arousal level on the change in positive affect. The interaction and main effects were not statistically significant, all *F*′*s* (2, 70) < 0.34, *p*′*s* > 0.56.

Follow up univariate two-way ANOVAs were run considering the main effect of the robot’s arousal level on self-disclosure. Data are mean ± standard deviation. There was a statistically significant main effect of the arousal level on the amount, *F* (1, 70) = 6.064, *p* = 0.016, *η* ^2^ = 0.080, and the intimacy of self-disclosure, *F* (1, 70) = 6.396, *p* = 0.014, *η* ^2^ = 0.084. Participants disclosed more to the low arousal robot, 27.25 ± 1.50, than to the high arousal robot, 21.95 ± 1.54. Moreover, the self-disclosures were also more intimate when disclosed to a low arousal robot, 29.14 ± 1.00 versus 25.50 ± 1.02.

Follow up univariate two-way ANOVAs were run considering the main effect of extraversion on self-disclosure. There was a statistically significant main effect of extraversion on the amount, *F* (1, 70) = 10.413, *p* = 0.002, *η* ^2^ = 0.129, and the intimacy of self-disclosure, *F* (1, 70) = 11.969, *p* = 0.001, *η* ^2^ = 0.146. Extraverts disclosed more (28.07 ± 1.52) and more intimate (29.804 ± 1.02) than introverts (21.13 ± 1.52 and 24.84 ± 1.01).

To check whether the participants perceived the extraversion of the robot as differently an independent-samples *t*-test was run. Participants rated the extraversion level of low arousal robot (4.03 ± 0.43) not significantly differently than the high arousal robot (3.97 ± 0.36), *t* (73) = 0.649, *p* = 0.518.

#### 5.3.4 Impact of similarity

Children could rate their similarity on a 5-point scale. On average participants felt slightly similar (3.6 ± 0.88) where only 1 participant felt completely dissimilar 1) and another 4 moderately dissimilar 2). The majority (31 participants) did not felt similar or dissimilar 3) or (26 participants) moderately similar 4). 12 participants felt completely similar.

Participants mentioned (dis)similarities in interest (e.g., “we both like football”), behavior (e.g., “he talked fast like me”), appearance (e.g., “the robot is smaller than me”), and interaction satisfaction (e.g., “I couldn’t always understand what the robot was saying”) as reasons for why they gave the rating they gave. Shared interests and similar behaviors were mostly mentioned as arguments for why children felt similar. The difference in appearance or a dissatisfaction about the interaction were mostly mentioned as arguments for why children felt not so similar. Interestingly enough, the participant who felt completely dissimilar mentioned that they “hated reading, dancing, and football”, all things the robot explicitly likes.

To explore the relationship between similarity and self-disclosure and positive affect change a Pearson’s product-moment correlation was run. The results are displayed in Table 6. There was a statistically significant small positive correlation between the feeling of similarity and the intimacy of the self-disclosures, *r* (74) = 0.28, *p* < 0.02 and a statistically significant moderate correlation between the feeling of similarity and perceived self-disclosure, *r* (74) = 0.35, *p* < 0.002. No statistically significant correlations were found between similarity and the amount of self-disclosure and positive affect change.

**TABLE 6:** Pearson’s correlation results of the feeling of similarity with self-disclosure (SD) and positive affect change.

	Amount of SD	Intimacy of SD	Perceived SD	Positive affect
Similarity	0.09	0.28^a^	0.35^b^	0.13

a Correlation is significant at the 0.05 level (2-tailed).

b Correlation is significant at the 0.01 level (2-tailed).

#### 5.3.5 Child-robot relationship formation

The positive affect scores from before and after the interaction were compared. Of the 72 included^3^ participants, 48 reported a higher positive affect afterwards, 19 reported a lower positive affect, and 5 reported no difference. A Wilcoxon signed-rank test determined that there was a statistically significant median increase (0.35) in positive affect from before (3.40) to after (3.75) the interaction with the robot, *z* = 4.6, *p* < 0.0005, *η* ^2^ = 4.6.

A multiple regression analysis was run to check if positive affect change can be predicted from self-disclosure and the social attractiveness of the robot. The multiple regression model statistically significantly predicted a positive affect change, *F* (2, 74) = 4.340, *p* = 0.017, *adjR* ^2^ = 0.108.

Finally, to explore the relationship between the different measurements for self-disclosure, i.e., the counted amount, intimacy, and perceived amount, the change in positive affect and the social attraction of the robot a Pearson’s product correlation was run. The results are displayed in Table 7. The amount of self-disclosure is only, but strongly, statistically significantly correlated with the intimacy of self-disclosure. The intimacy of self-disclosure is statistically significantly correlated will all other factors. The perceived amount of self-disclosure, the change in positive affect, and social attractions are all statistically significantly correlated with all factors except the amount of self-disclosure.

**TABLE 7:** Pearson correlation for different self-disclosure (SD) measurements, positive affect, and social attraction.

	Amount of SD	Intimacy of SD	Perceived SD	Positive affect
Intimacy of SD	0.81^a^			
Perceived SD	0.05	0.28^b^		
Positive Affect	0.10	0.26^b^	0.29^b^	
Social Attraction	0.15	0.34^c^	0.42^a^	0.26^b^

a Correlation is significant at the 0.001 level (2-tailed).

b Correlation is significant at the 0.05 level (2-tailed).

c Correlation is significant at the 0.01 level (2-tailed).

## 6 Discussion

### 6.1 Conversational interaction design patterns

With the aim to enable the robot to get acquainted with the child we designed and evaluated five conversational interaction design patterns (IDPs). The focus of the evaluation was to assess the effectiveness of the patterns to accomplish their respective objectives and facilitate children to self-disclose to the robot. The high response rates to the closed (98%) and pseudo-open questions (99%) show that the robot can use it to effectively elicit self-disclosure. The high response rate of the open questions (88%) confirms that most children want to (and do) freely explain themselves. This validates the pairing of closed and open questions (IDP1).

Although pseudo-open questions (IDP2) seemed unrestricted only in 5% of the cases an invalid (unspecified) answer was given. In most of those cases the robot did not recognize an answer and children ended-up choosing a different, but valid, answer via the touch-repair mechanism. This shows that with the right questions and preparation less restricted questions can be asked, validating IDP2.

The backchannel (IDP3) response rates show that substantive backchannels are the most effective (85%). Phrasal (51%) and non-lexical (21%) backchannels underperform. We believe it is mainly due to insufficient timing of the robot to include the backchannel utterances. The video footage suggests that children often thought the non-lexical or phrasal backchannels were the start of the next question and they simply waited until the robot continued.

The success rate of the touch-based recognition and repair pipeline (IDP4) shows that it is highly effective (100%) in processing valid responses. If speech recognition fails the first time it fails for 62% the second time. This indicates that switching to the touch modality is a necessary approach. 93% of the first touch recognition attempts succeed, validating IDP4.

The overall speech recognition performance (73% success rate), was the same as reported by (Kennedy et al., 2017). Most children had one or two unsuccessfully attempts and some children seemed to have more trouble talking to the robot than others. Speaking too softly or too soon were two of the most made mistakes by the children. Improving the implementation of the speech recognition system, e.g. with automatic volume boosting and a lower starting latency, would reduce these mistakes. When children are too verbose or say something unexpected are trickier problems. The solution here lies in improving the conversation management. For example, by asking the child to be brief when a verbose answer is detected.

Improving the robustness of the conversation is not only important for creating a more user-friendly experience. A correlation analysis show there a significant negative relationship exists between the amount of recognition errors and how comfortable the children feel in the conversation, how socially attractive the robot is, and how intimate the self-disclosures are. In the exit interview children seem to blame themselves, the robot, or both, for the mistakes. The recognition errors did not seem influence the positive affect change. Meaning that it did not inhibit child-robot relationship formation. However, it might have consequences on the long-term, because children initiate a more superficial relationship.

Another type of speech recognition error that is important to investigate are the times the robot understood the wrong thing. This happened in 8.7% of the cases. The most common response was that children adopted the incorrect answer as their own for follow-up questions. This confirms that children are receptive to suggestions or other influences by the robot to conform (Vollmer et al., 2018). It is highly desirable to implement a repair mechanism for these type of errors, otherwise the robot might personalize a future interaction based on incorrect information.

The results of the six-step turn-taking pattern (IDP5) show that in 96.5% of the cases a child responds concisely when they need to. This is confirmed by the average character count that furthermore shows that children, as intended, significantly elaborate more during the open follow-up questions. IDP5 makes it easy to understand for children how to talk to the robot.

### 6.2 ‘How to talk to me’ tutorial

A majority of the children (86%) only made up to two mistakes. This can only be partly explained by the effectiveness and efficiency of the conversational patterns. An important part is also the willingness of the children to cooperate with the robot. We observed a high amount of self-correcting behavior. If children spoke too soon, before the beep indicating the robot was ready to listen, they would repeated their answer. Or if the robot would ask the question again most children repeated their answer more loudly. A lot of mistakes were prevented by this behavior. To be able to cooperate children need to know how to so and they need a clear motivation of why.

During the exit interview we asked children how they would explain to other children how to talk the robot. With only a few expectations the children reiterated that you had to speak loud and clear and after the beep. The importance of these three things were highlighted during the tutorial. This shows that the tutorial succeeded in providing children with the ‘how’ to cooperate.

During the interview we asked children their reason for participating. The primary reason was the excitement to talk to a robot. Right after was the reason that the robot would help children in the hospital. This narrative was part of the recruitment letter and the tutorial. During the interview we asked children whether they were annoyed by mistakes made by the robot. 16 (22%) of the children felt annoyed at some point during the interaction. Most of the children who were not annoyed mentioned that because the robot was still learning, mistakes were to be expected and were not experienced as a problem. This narrative was only part of the tutorial. It is clear that for the majority of children the narrative of a robot that is training to be a care robot was a motivation to participate and to cooperate during the interaction. The narrative to manage children’s expectations about the abilities of the robot seems to have its effect on children’s error tolerance and for providing a ‘why’ to cooperate. A dedicated experiment exploring the effects of different narratives is needed to study this further.

### 6.3 The reciprocation strategy

The result of user study 1 showed that children disclosed more to a robot that explicitly liked what they liked. This is the opposite of hypothesis *H*1.1*a*, which we need to reject. The two main behavioral differences between both strategies are the two times (out of ten) the nuanced robot explicitly liked something else and the ‘that is my favorite too’ statements made by the explicit robot. By making these statements the robot highlights their similarity with the child (Bernier and Scassellati, 2010). This would explain why the child perceived the explicit robot as more similar (again in contrary to our hypothesis *H*1.1*b*). The statement furthermore is a positive affirmation of the child’s self-disclosure. It is plausible that this lowers the perceived risk for the next self-disclosure, making children feel comfortable to self-disclose more.

We furthermore have to reject the two remaining hypotheses *H*1.1*c* and *H*1.1*d* as well, because no differences of authenticity and enjoyment were found. Children in general rated the robot as not really authentic, but also not inauthentic. In hindsight it is more likely that the authenticity is more dependent on how authentic the content and the context of the conversation is, than a relative subtle difference in reciprocation strategy. The conversation was very linear. It cycles for ten times between the robot asking a question, the child giving an answer, and the robot following either reciprocation strategy giving a response back. In this simple implementation the robot had no clear personality in which the responses were grounded. There was no deeper motivation, other than the experiment, for the conversation. In other words, the content and context of the conversation were not very authentic to begin with, explaining the moderate scores. Children overall did strongly enjoy the conversation. It is plausible that the reciprocation strategy did not affect the enjoyment. More plausible however is that the novelty effect of chatting with a robot is what drove the enjoyment scores.

An open question is how durable the explicit reciprocation strategy is. The interaction was short, with a conversation that only consisted of ten back-and-fourths. Repeating the same statement over a longer period of time might have an adverse effect. Besides, once it is established that the robot is a trustworthy and comfortable conversational partner, the need for constant affirmation might be lower and the need to see something novel about the robot might increase. Then differences of factors like authenticity and enjoyment likely begin to show between both strategies. When that point is reached in a getting acquainted conversation is a question for future research. Explicitly liking what the child likes is, if anything, a good starting point for the child-robot relationship.

### 6.4 Robot arousal behavior profile

We have evaluated the behavior manipulations that were designed to elicit self-disclosures from introverted and extraverted children with a 2 × 2 between-subject user study. The results show no significant interaction effect of extraversion and the arousal level of the robot on self-disclosure. Instead, the results show that both extraverted and introverted children self-disclosed more and more intimately to the low arousal robot. We can therefore accept hypotheses *H*2.2*c* and *H*2.2*d*, but need to reject hypotheses *H*2.2*a* and *H*2.2*b*.

Results did confirmed hypothesis *H*2.2*e* showing that extraverted children significantly self-disclose more and more intimately than introverts. The known tendency of extraverted children to be more willing to self-disclose (Cuperman and Ickes, 2009) also holds for child-robot interaction.

Children did not rate the extraversion level of both robots as significantly different. One explanation is that the measurement was confounded by the dance activity. It was present in both robots, only the order (before or after the conversation) was different. Although the order difference made sure an extraversion matching effect could still occur, it is not likely that it did, because then we would have found the expected interaction effect. As a result, we conclude that the low and high arousal robot cannot be considered as being distinctly introvert and extravert and that no extraversion matching effect occurred.

An important lesson we take away from this is that it is difficult 1) to define what constitutes intro/extravert behavior for a robot, 2) to design concrete robot behaviors that are distinctly perceived as intro/extravert, and 3) to measure the perception of children regarding the extraversion of the robot and whether it matches. The question arises of whether we really need to create an intro/extravert robot to optimally facilitate self-disclosure, getting acquainted, or perform any other function? Especially since we are not the only ones having trouble (Robert, 2018).

The video footage revealed a number of leads why the low arousal robot is more effective to elicit self-disclosures. The arousal profile had no effect on the performance of the speech recognition. Participants did seem to be more ‘in sync’ with the low arousal robot. In sync means that the timing of, for example, the gestures, the questions, backchannels, and turn-taking is contingent with the speaking behaviors of the children. Whenever the timing was off children had to correct themselves more often to give answers and the robot would interrupt the child more. Being in sync is a defining feature for creating rapport (Gratch et al., 2007). More rapport leads to more self-disclosure (Duggan and Parrott, 2001; Vacharkulksemsuk and Fredrickson, 2012; Zhao et al., 2014). It also might be the case that the low arousal robot creates a more relaxed setting for the conversation. Good interviewers, especially in uncomfortable scenarios, create a relaxed setting explicitly and implicitly to elicit more self-disclosure (Wright and Powell, 2007; Morrison, 2013).

The lack of a extraversion matching effect could explain why we found no effect of the behavioral profiles on relationship formation (i.e., positive affect change). Relationship formation is also stimulated by better rapport and comfort when people get acquainted (Vacharkulksemsuk and Fredrickson, 2012). This would warrant a similar effect as with self-disclosure, which we did not observe. However, because exchanging self-disclosures is what facilitates relationship formation (Vittengl and Holt, 2000; Vacharkulksemsuk and Fredrickson, 2012) it is plausible that the interaction was not long enough for the behavior profile to have its effect.

### 6.5 Impact of similarity

Feeling similar to another is an accelerator for getting acquainted and relationship formation (Burleson, 1994; Montoya et al., 2008; Selfhout et al., 2009; Bernier and Scassellati, 2010). The robot only managed to create a sense of similarity for a half of the participants. 42% of the participants felt neither similar nor dissimilar. A minority (7%) felt dissimilar. Sharing interests was the most mentioned factor for feeling similar. Children who were unsure or felt dissimilar gave mostly metaphysical arguments about the difference between people and robots. Because the interaction mainly took place in the form of a conversation about hobbies and interests, it makes sense that sharing interests is an important factor for feeling similar. The topics that were discussed in the conversation were fairly generic and popular interests and hobbies. A question for future research is to explore whether personalizing the conversational topics to the specific interest of the children will increase the sense of similarity.

The sense of similarity did not statistically significantly correlate with the amount of self-disclosure, we therefore have to reject hypothesis *H*2.5*a*. Children overall seem to have a strong motivation to be heard by the robot. It is plausible that the need to give, at least a basic, answer to the robot’s questions is stronger, a least during the first 15 minutes, than factors like similarity. Similarity did correlate with the intimacy of the self-disclosure and the how much children thought they self-disclosed. It is plausible that feeling similar did reduces the risk for children to self-disclose more intimate things.

Furthermore, no significant correlation was found between similarity and positive affect change. We have to reject hypothesis *H*2.5*b*. Feeling similar and changes in positive affect are parallel processes of relationship formation that affect each other (Burleson, 1994). It is likely that, given the short time frame of the interaction, once the robot was established as a similar partner there was not enough time to significantly affect the change in positive affect. The more often and the longer the child will interact with the robot the more important experiencing similarities will likely become.

### 6.6 Child-robot relationship formation

In user study 2 we have replicated the experimental set-up of Vittengl and Holt (2000), who studied relationship formation within student-student dyads. Instead we studied child-robot dyads. Like the original study we observed a positive affect increase for most dyads. We can therefore accept hypothesis *H*2.6*a*. This is indicative of successful relationship formation (Baumeister and Leary, 1995). Furthermore, perceived self-disclosure and social attraction significantly predict the increase in positive affect. We can accept hypothesis *H*2.6*b* as well. It demonstrates that the processes for how children form relationships with each other and how they form relationships with robots is similar. Reciprocal self-disclosure is also a key factor for a successful child-robot relationship.

It is important to note that Vittengl and Holt (2000) and we in replicating the study set-up measured the perceived amount of self-disclosure. For the evaluation of the behavioral profiles we measured the actual amount of self-disclosure as well as the intimacy of the self-disclosures. With a Pearson’s correlation analysis we investigated the relationship between these different measures. Interestingly, the actual and perceived amount of self-disclosure did not correlate. The appears to be no relationship between the actual amount of self-disclosure and relationship formation. Such a relationship does exist for the intimacy of self-disclosure, which also correlates with the perceived amount of self-disclosure.

It could be that children are not able to reliable assess how much they actually self-disclose. More likely is that the more factual expressions are not considered as real self-disclosures (van Straten et al., 2022) and that they weigh the intimacy of what they said when they have to assess how much they self-disclosed. Thus, the perceived amount of self-disclosure is more of a reflection of the intimacy of the disclosures. Meaning that to further support child-robot relationship formation we need to focus on making it comfortable for children to self-disclose more intimate things. It is not only a matter of eliciting self-disclosure as much as possible. This has consequences for the design of the getting acquainted conversation and the conversations that follow. More work is needed to establish what an appropriate level of intimacy is. The work by Burger et al. (2017) provides a good starting point. The reciprocation strategy and conversational patterns need to be refined to appropriately respond to more intimate self-disclosures if we want them to be successful in a more long-term conversation.

### 6.7 Limitations

The focus of the first study was to pilot the two different strategies and the interaction as a whole. The results should be primarily treated as formative in nature rather than summative. Especially given the low sample size for a between-subjects study design.

The limitations of the second study lie in the assessment of the extraversion level of the robot, and the scoring of self-disclosure, in combination with the medium sample size for the complex study design. The extraversion scoring of the robot and the self-disclosure measures are not validated. Furthermore, the interaction design patterns are only jointly evaluated in this specific context. The study would benefit from additional validation of the used measures and a larger sample size.

Finally, although our designs were made with the pediatric population in mind, we did not validate the behavior designs with that population. A clinical evaluation is underway and will be reported in future work.

## 7 Conclusion

We studied how children and the robot get acquainted with one another and form a relationship. We have learned that similar key processes play a role in how children get acquainted and form relationships with other people as when they do so with robots. We replicated the experiment by Vittengl and Holt (2000) who studied student-student dyads getting acquainted only now with child-robot dyads. We found, like the original study, that reciprocal self-disclosure and social attraction are key predictors for the success of the relationship formation.

We furthermore found that relationship formation is not about the amount of self-disclosures, but about the intimacy of those disclosures. The more intimate the disclosures become the more children feel they disclose to the robot and that perception is a crucial step in the relationship formation process. It is important that children feel comfortable to self-disclose beyond the basic biographical facts.

We provide four concrete and reusable robot behavior designs that contribute to the goal of creating a comfortable interaction that elicits children to self-disclosure. The first is a set of five validated conversation interaction design patterns that enables a social robot to autonomously manage the getting acquainted interaction. We demonstrated that the patterns enabled the children to robustly self-disclose by asking a combination of closed, open, and pseudo-open questions. If speech recognition fails, the touch modality proved to be an effective repair mechanism. Due to the six-step turn taking pattern children quickly pick up on how to effectively talk to the robot. Results show that improvements can be made by refining the timing of backchannels, better supporting children to time their answers and signal them if they answer too verbosely, and by adding a repair mechanism for incorrectly recognized speech.

The second design element is a ‘how to talk to me tutorial’. It taught children to effectively talk to the robot, but most importantly it introduced the narrative of a robot that is still learning and is curious about the children. This narrative successfully grounded the errors that were bound to happen during the autonomous interaction. Children showed a lot of self-repair behavior and were more forgiving towards mistakes by the robot. This is helpful because the results show that the lower the number of mistakes, the more comfortable and intimate the conversation gets and the more socially attractive the robot is.

The third design element is a reciprocation strategy where the robot responds to the child’s self-disclosures by explicitly liking it and reciprocating with a matching anecdote. We demonstrated it elicits children to self-disclosure more and feel more similar to the robot. Results furthermore showed that children who felt more similar to the robot self-disclosed more intimately.

The fourth and final design element is an arousal behavioral profile for the robot. By manipulating the robot’s way of talking and moving more rapport and a more comfortable rhythm in the conversation can be created. It elicits children to self-disclose more and more intimately. It did not directly had an effect on relationship formation. We originally designed a high arousal profile for extraverts and a low arousal profile for introverts, but we did not find a matching effect. Instead we found that the configurations of the low arousal profile worked better for all children regardless of their extraversion trait.

With two rigorous user studies we increased our understanding of how children get acquainted with and form a relationship with a robot. We demonstrated that our designs successfully equip the robot to autonomously manage a getting acquainted conversation and foster the child-robot relationship. Although there is still much to be learned and improved, we hope that we made a solid step towards a successful deployment of a socially assistive robot for children who could really use one.

## Data Availability

The raw data supporting the conclusions of this article will be made available by the authors, without undue reservation.

## Footnotes

1 Weizenbaum originally set out to demonstrate the superficiality of communication between people and computers, but was surprised by how much people anthropomorphize ELIZA and by the intimacy of the things people self-disclosed to ELIZA (Weizenbaum, 1976).

2 The software is available here: https://github.com/HeroProject/HeroGoalAgent.

3 3 outliers were removed.
